# 폐경증상 관리를 위한 중년 여성의 신체활동 참여 경험: 현상학적 연구

**DOI:** 10.4069/kjwhn.2023.06.15

**Published:** 2023-06-30

**Authors:** Hee Jung Cho, Sukhee Ahn

**Affiliations:** College of Nursing, Chungnam National University, Daejeon, Korea; 충남대학교 간호대학

**Keywords:** Menopause, Middle aged, Physical activity, Qualitative research, Women, 폐경기, 중년, 신체활동, 질적 연구, 여성

## Abstract

**Purpose:**

The purpose of this study was to comprehensively understand and describe the meaning of physical activity for managing menopausal symptoms in middle-aged women.

**Methods:**

This study targeted middle-aged women with menopausal symptoms who participated in regular exercise at least three times a week for more than 12 weeks. Nine participants were individually interviewed via in-depth face-to-face interviews, and participatory observation was also employed. Colaizzi’s phenomenological qualitative research method was applied for analysis.

**Results:**

Participants were asked, “What does it means to participate in physical activity at this time of your life?” Fourteen codes, six themes, and three theme clusters were derived for the meaning of physical activity for managing menopausal symptoms in these middle-aged women. The six themes were “reviving the exhausted body and mind,” “being free from the yoke of pain,” “being settled in life,” “finding oneself and becoming altruistic,” “striving while anticipating change,” and “equipping the body and mind.” The three theme clusters were “overcoming my past pain,” “taking the initiative for today’s life,” and “moving towards new change.”

**Conclusion:**

The narratives revealed that physical activity allowed women to overcome menopausal symptoms, the burden of relationships, and stress, thereby enabling them to make positive changes in their lives and have expectations for the future. Thus, physical activity was a positive force in a healthy menopausal transition for women with menopausal symptoms. The findings of this study can be used to encourage physical activity in peri-menopausal women and to develop physical activity programs for managing menopausal symptoms.

## Introduction

신체활동은 일상 생활에 밀접하게 관련되어 있으면서 건강상의 많은 유익을 주는, 건강 증진을 위한 대표적인 방법이다. 신체활동의 부족은 모든 질병에 대한 위험을 증가시킬 뿐만 아니라 근육과 폐, 심장 기능을 약화하고 비만을 비롯한 각종 성인병을 초래하는 원인이 되며[1], 폐경 후 중년 여성의 우울 증상에 영향을 미쳐 여성의 건강관련 삶의 질을 떨어뜨린다[2].

선행연구에 의하면 주2회 50분씩 총 10주–12주간의 신체활동만으로도 폐경기 여성의 체중과 체지방이 변화되며, 수면의 질을 개선하고 요실금 증상과 우울 완화에 효과가 있는 것으로 나타났다[3,4]. 그러나 현재 한국 중년 여성의 신체활동 참여도는 많이 낮을 뿐만 아니라[5], 계속해서 신체 활동량이 더 낮아지고 있는 실정이다[6]. 특히 중년 여성의 신체 활동량은 남성보다 적은 것으로 나타났는데[7], 남성에 비해 여성은 가사 활동량이 더 많은 반면 여가 활동량은 상대적으로 적은 것으로 조사되고 있다[8]. 이에 한국 보건복지부는 신체활동 지침을 통해 한국 성인은 근력운동을 일주일에 2회 이상 수행하고, 일주일에 150분 이상의 중강도 유산소 신체활동 또는 일주일에 75분 이상의 고강도 신체활동을 수행할 것을 권장하고 있으며[9], 이와 함께 본인의 자각 정도에 대한 기준을 휴식할 때의 자각 강도인 1에서부터 본인이 수행할 수 있는 가장 높은 강도인 10까지 정하여 제시함으로써 실생활에서 누구나 쉽게 신체활동에 적용할 수 있도록 하고 있다.

폐경은 중년 여성이 경험하는 정상적인 생의 과정에 속하지만 여성의 삶에서 생리적·정신적·사회적 위기로 작용할 수 있다. 이 시기에 여성은 피로와 수면장애, 안면홍조, 두통 등의 다양한 폐경증상과 함께 성적인 감정과 태도의 변화, 관계의 변화 등 다방면의 변화를 경험하게 된다[10]. 그리고 이것은 폐경기 여성의 삶에 직접적으로 부정적인 영향을 미치며 노화에 대한 불안을 높이는 요인이 된다[11,12]. 현재 한국 여성의 평균 폐경 나이는 49.9세로 보고되고 있으며[13], 일반적으로 폐경증상은 폐경 4–6년 전에 시작되어 몇 년간 계속되는데[14], 이 기간 동안 폐경증상은 지속적으로 여성의 삶의 질을 떨어뜨리는 원인이 될 뿐만 아니라 그대로 방치할 경우 폐경 이후 대사증후군, 이상지질혈증 등의 이차적 질병 유병률이 증가하게 된다[15,16]. 그러므로 폐경기 여성을 위한 보다 적극적인 폐경증상 관리가 요구되는 실정이다.

신체활동은 폐경기 여성의 체중 및 체지방과 수면의 질을 개선하고 폐경증상 완화에 효과가 있어[3,4], 폐경증상 관리를 위한 생활 속 실천 전략으로 활용하기에 적합하다. 따라서 이를 구체적으로 적용하기 위해서 폐경기 여성의 신체활동에 대한 의미를 자세히 살펴볼 필요가 있다.

그러나 지금까지 중년 여성을 대상으로 한 신체활동 관련 연구는 대부분 신체활동의 영향 및 효과에 대한 연구로[17-19] 폐경증상에 대해서 직접적으로 고려하지 않은 경우가 많고, 대상이 중년 여성 또는 폐경기 여성으로 모호하게 사용되고 있어서 폐경증상 관리를 위한 신체활동에 대한 이해가 명확하지 않은 실정이다. 또한 기존의 질적연구에서도 요가나 수영, 댄스 등 특정 활동에 대한 경험에 국한되어 있어서[20-22], 관련 현상에 대해 전체적인 맥락에서 총체적으로 이해하기에 한계가 있다.

실무에서 필요로 하는 대상자 중심의 간호를 제공하기 위해서는 현상에 대한 정확한 이해가 선행되어야 하며, 연구에 대한 결과가 실무에서 잘 적용되고 있는지 계속적으로 확인하는 과정이 수반되어야 한다. 특히 폐경증상과 관련된 현상은 현상을 실제 경험 그대로 이해하고 해석하려는 노력이 필요한데, 질적 연구 중 Colaizzi [23]의 현상학적 분석 방법은 전체 참여자의 진술을 구체적으로 반영함으로써 도출해 낸 공통적인 속성을 토대로 하여 현상에 관련된 경험의 본질을 총체적으로 이해하기에 적합하다.

그러므로 본 연구는 Colaizzi [23]의 현상학적 분석 방법을 적용하여 폐경증상이 있는 중년 여성을 대상으로 그들의 실제 삶을 탐색함으로써 그들은 신체활동에 참여하면서 어떤 경험을 하고 있으며, 그들은 이 경험을 어떻게 해석하는지, 그들에게 이 경험이 가지는 본질적인 의미가 무엇인지를 그들의 관점에서 이해하여 기술하고자 한다. 이로써 폐경증상 관리를 위한 신체활동 의미에 대한 이해를 돕고자 하며 궁극적으로는 폐경기 여성의 삶의 질을 높이고 성공적인 폐경 이행과 건강한 노년기로의 진입을 도울 수 있기를 기대한다. 따라서 본 연구의 목적을 위하여 살펴볼 구체적인 연구 문제는 “폐경증상 관리를 위한 중년 여성의 신체활동 참여 경험의 본질적 의미는 무엇인가?”이다.

## Methods

Ethics statement: This study was approved by the Institutional Review Board of Chungnam National University (202205-SB-057-01). Informed consent was obtained from the participants.

### 연구 설계

본 연구는 폐경증상 관리를 위한 중년 여성의 신체활동 참여 경험의 의미를 총체적으로 이해하고 기술하기 위하여 Colaizzi [23]의 현상학적인 분석 방법을 적용한 질적 연구이다. 본 연구는 SRQR (Standards for Reporting Qualitative Research) 가이드라인(https://www.equator-network.org/reporting-guidelines/srqr)을 준수하여 기술하였다.

### 연구 참여자 선정과 특성

폐경증상 관리를 위한 중년 여성의 신체활동 참여 경험을 심층적으로 탐색하기 위하여 현재 폐경증상 관리를 위해 신체활동에 참여하고 있는 중년 여성을 대상으로 하였다. 본 연구에서 신체활동이라 함은 여성이 폐경증상 관리를 위해 규칙적으로 수행하는 운동을 의미한다. 본 연구에서는 풍부한 경험을 제공해 줄 수 있는 참여자를 선정하기 위해 목적적 표본추출 방법을 적용하였다. 한국 여성의 평균 폐경 나이가 49.9세이며[13] 일반적으로 폐경증상은 폐경 4–6년 전에 시작되어 몇 년간 지속된다는 것을 근거로[14], 폐경증상을 가장 많이 경험하는 연령층에 해당하는 만 45세–60세의 중년 여성을 대상으로 선정하였다. 즉, 폐경증상 관리를 위하여 1회당 50분 이상씩 주 2회 이상, 최근 12주 이상 운동에 참여하고 있는 폐경기 여성을 대상으로 하였으며, 이는 신체활동을 주 2회 50분씩 10주 실시했을 때 여성의 폐경증상이 의미 있게 변화된 선행연구에[4] 근거한다. 또한 본 연구에서는 폐경증상 관리를 위한 신체활동 경험에 초점을 두기 위하여 여성 호르몬을 처방 받아 복용 중인 여성은 대상자 선정에서 제외하였다.

따라서 본 연구의 취지에 가장 적합한 참여자를 모집하기 위해서 서울시 소재 한 피트니스 센터의 group exercise (GX) 프로그램에 참여하고 있는 회원을 대상으로 모집하였다. 센터 관리자 동의 하에 회원 대상 모바일 대화방에 모집공고문을 배포하여 1차로 모집하고, 이들 중 폐경증상 관리를 위한 대상자임을 본 연구자가 확인한 후 본 연구의 참여자로 선정하였다. 최종적으로 본 연구에 참여한 연구 참여자 수는 더 이상 새로운 진술이 나타나지 않는 상태인 포화에 이른 숫자로 총 9명이며 일반적 특성은 Table 1에 제시하였다. 연구 참여자의 나이는 만 52세에서 59세로 평균 나이는 55.8세이며, 참여자들은 모두 폐경이 된 상태로 평균 폐경 나이는 51.4세이다. 본 연구의 참여자들은 수면장애와 열성 홍조, 우울과 피로, 건조증, 두통 등 두 가지 이상의 다양한 폐경증상을 경험하고 있었다. 또한 참여자들의 평균 신체활동 참여 기간은 3.8년(4개월–10년)이었으며, 각각 GX 강좌 프로그램인 요가, 에어로빅, 줌바댄스 중 한 가지 이상의 운동을 하고 있었고 3명은 그 외에 수영, 골프, 밸리댄스 등을 추가로 하고 있었다.

### 연구자 준비

본 연구의 연구자들은 질적연구의 연구 도구로써 연구의 전 과정에 걸쳐 성찰적 견지를 유지하면서 각 개인의 선이해와 편견을 배제하고 정확한 결과를 도출하기 위해 노력하였다. 또한 연구자들은 자료의 수집과 분석, 결과 도출과 마지막 최종기술에 이르기까지 자료의 의미에 대한 정확한 이해를 위해 함께 논의하며 고심하였다.

자료수집을 담당한 본 연구자는 GX 프로그램에 회원으로 등록하여 각 프로그램에 자유롭게 참여하여 그들을 가까이에서 관찰하였으며 참여자들을 더 깊이 있게 이해하기 위하여 평소 신뢰 관계 형성을 위하여 노력하였다. 또한 현재 폐경증상을 관리하기 위하여 요가 프로그램에 참여하여 규칙적으로 신체활동을 실천하고 있으며, 중년 여성을 대상으로 하여 자기관리를 위한 신체활동 교육 프로그램을 진행하고 있다. 본 연구자는 대학원 박사과정에서 심층 면담에 대한 훈련과 현상학적 연구 방법을 포함한 질적 연구 방법에 대하여 숙지하고 훈련하는 충분한 준비기간을 가졌으며, 워크숍과 강좌와 서적을 통하여 질적연구 방법을 이해하고 습득한 기술을 본 연구에 활용하였다.

### 자료 수집

본 연구를 위한 자료수집은 2022년 9월–11월의 3개월 동안 심층 면담과 참여관찰을 통하여 수행하였다. 심층 면담을 위한 주요 질문은 “신체활동 참여는 귀하에게 어떤 의미인가요?”였으며, “귀하의 폐경증상 관리를 위한 신체활동 경험에 대해 자유롭게 말씀해 주시겠어요?”로 시작하여, “신체활동 참여는 귀하의 삶에 어떠한 영향을 주었나요?”를 추가하면서, 참여자의 경험을 자유롭게 끌어내고자 하여 최대한 질문 수를 줄이고 상황에 따라 자연스럽게 질문을 이어가는 형식으로 하였다. 본 연구자는 적절하고 충분한 자료를 수집하기 위하여 센터의 회원으로 가입한 후 참여자들과 함께 GX 프로그램에 참석하여 참여자들을 관찰함으로써 그들의 경험을 좀 더 깊이 있게 이해하고자 노력하였다. 또한 본 연구자는 요가와 줌바댄스 프로그램에 참여하여 참여자들이 평소 어떤 폐경증상을 경험하고 있는지를 조사하고, 그들의 얼굴 표정과 태도, 주고받는 대화를 관찰하여 자료분석에 활용하였다. 면담 장소는 피트니스 센터와 가장 가까운 카페에서 면대면 면담으로 진행하였다. 자유롭게 이야기할 수 있도록 참여자 외에 면담에 같이 동참한 사람은 없었으며, 약 일주일 이상의 자료 수집 간격을 두면서 자료 수집과 동시에 자료의 분석을 함께 진행하여 다음 면담에 반영하였다. 심층 면담은 1회 진행하였으며 1시간 30분–2시간 동안 이루어졌다. 누락을 방지하기 위하여 참여자의 동의 하에 녹음을 진행하고, 면담 분위기와 비언어적 표현 등 특이 사항은 분석에 활용하기 위하여 현장 노트에 바로 기록하였다. 또한 떠오르는 아이디어나 생각은 바로 메모하고 이해되지 않는 내용은 추후 신체활동 프로그램에서 만나 확인하였다. 면담 진행은 더 이상 새로운 자료가 나오지 않는 상태인 자료의 포화 상태에 이를 때까지 하였고, 그날 바로 본 연구자가 직접 녹취하였으며, 필사한 본 파일을 모바일 폰으로 참여자에게 전송하여 참여자가 면담 내용을 직접 확인하게 하여 면담의 내용이 사실과 일치하는지 확인하였다.

### 자료 분석

본 연구의 자료 분석은 Colaizzi [23]의 현상학적 방법을 적용하였다. 첫 번째 단계에서는 연구자가 참여자의 신체활동 참여 경험의 의미를 이해하기 위해 전체적인 느낌을 얻고자 노력하며, 녹음된 면담 내용을 반복하여 들으면서 참여자가 진술한 그대로를 옮겨 기록하였다. 두 번째 단계에서는 필사본을 여러 번 읽으면서 본 연구의 관심 현상에 부합되는 부분에 밑줄을 치며 의미 있는 진술을 도출하였다. 세 번째 단계에서는 의미 있는 진술로부터 324개의 좀 더 일반적인 형태로 재진술하였다. 네 번째 단계에서는 의미 있는 진술과 재진술에서 147개의 구성된 의미를 도출하였다. 다섯 번째 단계에서 구성된 의미를 분류하여 코드, 주제, 주제묶음으로 조직하고 이 조직의 내용과 필사본이 같은 내용의 맥락을 유지하는지 확인하였다. 이후 공동연구자와 함께 면담 내용으로부터 참여자들의 공통적인 속성을 적합하게 도출하였는지에 대해 재확인하고 반복적으로 검토하면서 최종적으로 14개의 코드와 6개의 주제, 그리고 3개의 주제묶음으로 조직하였다. 여섯 번째 단계인 최종적인 기술에서는 연구 결과에 대한 연구자 간 충분한 논의를 거쳐 참여자들의 신체활동 참여 경험에 대해 총체적으로 통합하여 기술하였다. 마지막으로 참여자를 만나 분석 결과가 그들의 경험과 일치하는지 확인하였으며, 참여자의 사정으로 직접 만날 수 없는 경우에는 전화 통화로 동일한 과정을 거쳤다.

### 연구의 엄밀성

본 연구의 신뢰도와 타당성을 확보하기 위하여 Guba와 Lincoln [24]이 질적연구 평가 기준을 위하여 제시한 과학적 연구의 진실성을 따르기 위해 노력하였다. 첫째, 사실적 가치(truth value)를 확보하기 위하여 본 연구자는 사전에 참여자와 함께 활동에 참여함으로써 적합한 대상자 선정을 위해 노력하였다. 본 연구자는 요가와 줌바댄스 프로그램에 참여하면서 참여자들이 평소 어떤 폐경증상을 경험하고 있는지 관찰하여 가장 적합한 대상자 선정에 참고하였으며, 신체활동에 참여하는 동안 참여자들의 행동 뿐만 아니라 얼굴 표정과 태도 및 신체활동 후 주고받는 대화를 관찰하여 자료분석에 반영하였다. 또한 진정성 있는 경험을 끌어내기 위해 평소 신뢰 관계 유지를 위해 노력하였으며, 본 연구의 취지에 맞는 가장 적절하고 충분한 자료를 수집하고자 노력하였다. 면담 내용에 대한 타당성을 확보하기 위해 면담 내용을 기록한 필사본을 각 참여자에게 보여주고 면담의 내용이 사실과 일치하는지 확인하였으며, 일대일 만남 및 전화통화를 통해 본 연구의 결과가 참여자들의 경험을 잘 나타내 주고 있는지에 대해 확인하는 과정을 거쳤다. 둘째, 적용성(applicability)을 위하여 포화에 이르기까지 충분한 자료를 수집하고, 분석된 연구의 결과를 심층적으로 기술함으로써 참여자들의 신체활동 경험의 의미를 잘 전달하고자 노력하였다. 셋째, 일관성(consistency)을 위하여 미리 준비된 면담을 위한 질문을 중심으로 심층 면담을 진행하고 본 연구의 자료 분석 기법인 Colaizzi [23]의 자료 분석 과정의 절차를 따랐으며, 모든 과정은 기록하고 분석 결과는 질적연구의 경험이 많은 간호학 박사 2인의 의견을 수렴하였다. 이 과정에서 일부 모호하게 도출된 주제 및 범주를 수정하였고, 연구의 목적에 맞게 결과가 바르게 기술되었는지에 대해 검토한 후 내용이 부적절한 부분은 보완하였다. 넷째, 중립성(neutrality)을 확보하기 위하여 본 연구의 참여자를 제외하고 현재 폐경증상 관리를 위해 신체활동에 참여하고 있는 중년 여성 2명을 임의 선정하여, 본 연구의 결과가 폐경관리를 위한 중년 여성의 신체활동 경험을 잘 나타내고 있는지에 대해 확인하는 과정을 거쳤다. 또한 폐경증상 경험에 대한 연구자의 개인적 의견에 대한 지속적인 괄호 치기(bracketing)와 자기성찰을 위해 성찰일지를 활용하였으며, 연구자의 선경험을 판단 중지하고 그들의 경험을 있는 그대로 반영하여 현상에 대한 경험의 본질을 찾고자 노력하였다.

## Results

폐경증상 관리를 위한 중년 여성의 신체활동 참여 경험의 의미는 14개의 코드, 6개의 주제, 3개의 주제묶음으로 도출되었다(Table 2). 최종적으로 도출된 3개의 주제묶음인 ‘과거의 고통에서 벗어나기’, ‘오늘의 삶에서 주도권 잡기’, ‘새로운 변화를 기대하며 나아가기’에 대한 의미 있는 진술은 다음과 같다.

### 주제묶음 1. 과거의 고통에서 벗어나기

이 주제묶음에는 ‘소진된 몸과 마음 살리기’, ‘고통의 굴레에서 벗어나기’가 포함되었다. 참여자들은 폐경증상으로 인해 몸과 마음이 소진된 상태에서 남은 삶을 살아내기 위해 신체활동에 참여하였고, 관계에 대한 집착과 스트레스 등으로 인한 고통에서 벗어나는 경험을 한 것으로 나타났다. 참여자들은 폐경증상이 조금씩 나아지고, 타인과의 관계에 집착하던 것에서 자유로워지고, 그동안 쌓였던 스트레스를 풀어내면서 고통의 굴레에서 벗어나게 되었고 그러한 고통을 지나간 과거로 인식하고 있었다. 이는 참여자들이 신체활동을 통하여 그동안 참여자들을 옥죄고 있었던 고통을 극복하게 되었다는 것을 의미한다.

#### 주제 1: 소진된 몸과 마음 살리기

참여자들은 폐경증상으로 인해 몸과 마음의 건강상태가 일상 생활을 유지하기 힘들 정도로 나빠졌다고 하였다. 온몸을 타고 뜨겁게 달아오르는 열감으로 수면장애를 경험하기도 하였고, 직장생활 뿐만 아니라 일상생활이 어려울 정도로 우울감과 무력감으로 시달렸다고 하였다. 따라서 참여자들이 신체활동을 선택한 것은 남은 인생을 살아내기 위한 마지막 몸부림이라고 하였다. 참여자들은 대부분 두 가지 이상의 폐경증상으로 인해 고통받았었는데, 각자 자기에게 맞는 신체활동을 찾아 꾸준히 참여하게 되면서 조금씩 그 증상이 상당히 개선되었을 뿐만 아니라, 남아 있는 증상에 대해서도 스스로 자신의 몸을 조절할 수 있겠다는 자신감이 생겼다고 하였다.

“관리하면 본전이고 관리를 안 하면 완전히 그냥 바닥을 치는 거지. 운동 안 하면 몸도 완전 바닥이고 직장생활도 유지가 안 되니까. 지금 이 나이에는 운동 안 하면 직장을 그만둬야 할지도 모를 정도로 몸이 안 좋잖아요.” (참여자 2)

“나는 폐경증상이 발바닥에 있었는데, 한겨울인데도 발바닥이 너무 뜨거워서 견디지 못하는 거야. 그래서 자다가 샤워기로 물을 뿌려서 발바닥 식히고, 앉아서 잤어. 이제 내가 살아야겠다 싶어서 바로 집 앞에 요가를 등록했죠.” (참여자 3)

“폐경 후 안구건조증, 질건조증, 구강건조증 다 같이 왔죠. 내 팬티 안에서 냄새가 나는데… 고통이 말도 못해. 오만 약 다 쓰고 뭐. 그런다고 좋아지는 거 아니야. 내 몸 자체에 호르몬 밸런스 때문에 그런 거니까. 운동 안 하면 어깨도 결리고 목도 쑤셔” (참여자 4)

“불면증이 와서 밤을 꼴딱 새는 거는 허다하고, 몸이 활활 타 오를 때는 순간적으로 온몸에 비 오듯이 땀이 나서 방금 샤워하고 나왔는데도 수건으로 닦고 있는데 그 자리에서 바로 땀이 올라와요. 운동하면 잠도 더 잘 자고 운동으로 열을 빼고 나면 좀 나아요.“ (참여자 9)

#### 주제 2: 고통의 굴레에서 벗어나기

참여자들은 신체활동에 참여하면서 다른 사람보다는 자신에게 더 집중하게 되었고, 자연스럽게 집안과 남편에 대한 집착의 굴레에서 서서히 벗어나게 되었다고 하였다. 그동안 누군가의 며느리이자 아내로 또 엄마로 열심히 살아왔지만 늘 다른 사람에게 집착하는 듯한 자신이 싫고 스스로 갑갑하게 느껴졌는데, 생전 처음으로 이제까지는 느끼지 못했던 해방감을 경험하게 되었다고 하였다. 참여자들은 음악에 맞춰 몸을 흔들고 요가를 하는 등 각자 자신이 좋아하는 신체활동을 마음껏 하면서 회사 일과 생활에서 오는 각종 스트레스와 부정적인 생각을 모두 풀어낸다고 하였다. 그래서 올 때는 축 처진 모습으로 왔다가 끝나고 집으로 갈 때는 한결 가볍고 밝은 모습으로 돌아간다고 하였다.

“운동하면서 집안에 대한 집착, 남편에 대한 집착이 많이 없어지는 것 같아. 남편이 늦게까지 안 들어오면 전화를 하게 되는데 내가 운동하러 가면 그걸 잊어버려요. 근데 집에만 있으면 한 시간이 지나면 약을 먹겠다 뭐… 체크를 하게 되거든요.” (참여자 5)

“지금 생각하면 그때 왜 그렇게 바보같이 살았는지 모르겠어. 운동하고 나서는 신랑이 못되게 해도 ‘나는 운동하니까 내가 조금 봐준다.’ 그러니까 싸울 일도 줄고, 남편이 늦게 들어오면 언제 오는지 기다리는 게 서로 힘든 건데, 그런 거에서 해방되니까 너무 좋아.” (참여자 8)

“운동하고 있는 동안에 시름이나 걱정거리 그런 게 다 사라지지. 힘들 때! 회사 일에서나, 어떤 일이 닥쳤을 때 힘들잖아요… 운동하면서 스트레스 풀고” (참여자 8)

“운동하면 부정적이었던 생각이 긍정적으로 변하는 거죠. 스트레스를 날려버리고 생각이 정리되는 느낌도 좋고, 암튼 스트레스 쌓인 걸 운동하면서 다 푸니까 운동하고 나올 때는 몸도 마음도 엄청 시원하죠.” (참여자 9)

### 주제묶음 2. 오늘의 삶에서 주도권 잡기

이 주제묶음에는 ‘삶 속에 자리잡음’, ‘자기를 찾고 이타심이 생김’이라는 주제가 포함되었다. 참여자들은 자기에게 잘 맞는 신체활동을 발견하고 그 재미에 푹 빠지게 되면서 신체활동에 참여하는 것을 중심으로 생활한다고 하였다. 또한 참여자들은 온전히 자신에게만 집중하는 시간을 통해 그동안 무관심했던 생활 속 변화를 깨닫게 되었고, 마음의 폭이 넓어지면서 다른 사람을 이해하고 용서하는 마음의 여유가 생겼다고 하였다. 이로써 참여자들은 조금씩 자기 삶에 대한 주도권을 회복해 가고 있는 것으로 나타났다.

#### 주제 3: 삶 속에 자리 잡음

참여자들은 그동안 여러 번의 시행착오를 거치면서 각자 자기에게 잘 맞는 신체활동을 찾게 되었는데 이제는 몸 상태에 따라 스스로 활동량을 조절할 수 있을 뿐만 아니라 무엇을 어떻게 해야 할지에 대한 갈등을 더 이상 하지 않아서 생활이 만족스럽다고 하였다. 또한 참여자들은 신체활동에 참여하는 것이 너무 재미있어서 시간이 지날수록 더 깊이 빠져든다고 하였다. 어느덧 신체활동에 참여하는 것이 삶의 일부이자 필수가 되었고 가끔 참여하지 못할 때에는 불안을 느끼기도 하지만, 오히려 이러한 감정은 신체활동을 지속하는 데 도움이 된다고 하였다. 참여자들은 신체활동 참여를 일상의 모든 선택에서 가장 우선 순위에 두고 있었는데, 시간을 맞추기 어려울 때도 센터에 와서 샤워라도 하고 가면 신체활동에 참여한 것과 같은 기분이 느껴지고 마음에 위안이 된다고 하였다.

“나는 요가가 좋아. 기구의 도움을 받지 않고 내 몸만 있으면 할 수 있으니까. 나는 내 성격이 몸을 흔드는 댄스 같은 건 못 하겠어. 음악에 맞춰서 뭔가를 하는 게 나하고는 안 맞아. 음악 소리가 나서 심장이 쿵쾅거리고 펌프질 하는 거 같아.” (참여자 3)

“요가보다는 나는 줌바가 좋아. 몸을 흔들어야 좋아… 스트레스 받지 말고 해야 해. 자기한테 맞는 거를 찾으면 돼.” (참여자 4)

“너무 행복해. 운동 안 하면 못 살 것 같아. 운동에 완전 진심이지. 거의 한 3년을 이렇게 해보니까 거의 중독된 것처럼 이제 안 하면 불안하고 계속해야 되겠다는 생각이 들어.” (참여자 8)

“음악을 들으면서 하니 신나잖아요. 나에게 맞아요. 에어로빅은 이제 내 다리가 허락하는 한은 하고 그 다음은 걷기라도 할거예요. 운동이 나의 삶의 일부가 된 것 같아요. 이제 운동 안 하면 진짜 미칠 것 같아. 필수인 것 같아요.” (참여자 6)

“빨리 해야 내가 운동 갈 수 있으니까… 대충 하고 오는 거지. 저녁 11시에 끝나도 여기 와서 씻고 집에 가는 것이 운동이라고 생각하지. 일단 여기 오는 거야. 도장을 찍어요.” (참여자 5)

“나는 어떤 것보다도 운동이 우선이야. 운동을 오전에 하고 12시에 미용실 열어. 운동 끝나고 와서 챙겨 먹고 8시까지 일하지. 기본 8시간은 일해. 나는 이 업도 중요한데 우선 순위로 건강을 위해서 오전은 운동에 다 투자해.” (참여자 7)

#### 주제 4: 자기를 찾고 이타심이 생김

참여자들에게 신체활동에 참여하는 시간은 오로지 자기자신에게만 집중하는 시간이라고 하였다. 따라서 참여자들은 자신의 내면을 들여다보면서 조용히 집중하는 성찰의 시간을 가지게 되었고, 자신이 너무 소중하게 느껴지면서 이제부터는 자신의 인생을 스스로 통제할 수 있겠다는 자신감을 회복하게 되었다고 하였다. 참여자들은 그동안 무기력했던 몸과 마음이 살아나고 인생을 즐길 수 있는 감정을 되찾게 되었는데, 점점 우울한 감정이 사라지고 건강과 생동감을 느끼게 되었고, 신체활동에 참여하기 위해 오가는 거리의 풍경도 매번 다르고 새롭게 느끼게 된 것으로 나타났다. 또한 그동안 다른 사람과의 관계에서 속이 많이 좁았었다는 것을 깨닫게 되었고 이제는 폐경기를 겪어야 하는 같은 여자로서 시어머니에 대한 측은지심도 생기고 늘 여기저기 아프다고 하셨던 친정 어머니의 심정도 이해된다고 하였는데, 이는 신체활동에 규칙적으로 참여한 이후 몸이 회복되면서 점차적으로 마음의 폭이 넓어지게 된 것을 의미한다.

“누구를 보여주기 위한 것도 아니고, 어느 날 폐경이 오고 몸이 안 좋아지면서 운동을 하게 되었는데, 그러면서 나를 객관적으로 보게 되었고 오히려 나를 찾고 사랑하게 된 거지.” (참여자 3)

“나는 운동할 때 나한테만 집중되는 느낌이 좋아요. 다른 건 신경 안 써도 되고 운동만 하면 되잖아요. 운동하면서 내 생각, 계획 같은 것들을 정리를 한 번 해봐요. 내 인생을 통제할 수 있다는 자신감이 들어요.” (참여자 9)

“요가를 함으로 해서 내가 규칙적으로 나가야 하는 일이 생기니까 삶의 활력이 될 수 있는 거예요. 그 짧게 걸어가는 5분 사이에 매번 다른 사람들! 하늘도 틀리고 공기도 틀리고 사람들도 틀리고.” (참여자 3)

“제가 운동하니까 여러모로 좋아요. 건강해지고 생기가 있고 일할 때 활력이 생긴 것 같아요. 특히 내가 밤에 일하는데... 운동을 하니까 이제 축축 처지는 것도 없어지고∙∙∙” (참여자 6)

“시집왔을 때 시어머니가 폐경기였는데 지금 생각해보니까 그 양반이 그때 힘드셨겠다 싶어. 처음에는 약이 오르고 왜 나한테만 저럴까? 그랬는데 지금은 측은지심, 마음의 여유가 생긴 거고. 그 땐 내 몸이 죽겠는데 그게 눈에 들어오겠어요?” (참여자 1).

“옛날에 우리 엄마가 일어날 때 ‘아이구... 여기저기 아픈 데도 많다’ 그랬거든. 이제 그 심정이 이해돼. 지금은 운동으로 극복했지만 내가 그 나이가 돼 보니까 쉽게 안 일어나지더라.” (참여자 5)

### 주제묶음 3. 새로운 변화를 기대하며 나아가기

이 주제묶음에는 ‘변화를 기대하며 노력하기’, ‘몸과 마음 갖추기’가 포함되었다. 본 연구의 참여자들은 건강과 젊음을 유지하고 싶어서 신체활동에 좀 더 적극적으로 참여하면서 체중을 관리하고 몸매도 가꾸기 위해 노력한다고 하였다. 또한 참여자들은 곱고 건강하게 나이 들어가기를 간절히 원하고 있었으며, 노년에도 할 수 있는 적합한 신체활동을 구상하면서 몸과 마음을 계속 갖추어 나간다고 하였다. 이것은 앞으로의 삶이 점점 더 나아질 것을 기대하며 현실을 극복해 나가고자 하는 참여자들의 심리를 나타낸다.

#### 주제 5: 변화를 기대하며 노력하기

참여자들은 늙어가는 자신의 모습이 계속 신경 쓰이고 때로는 불안을 느낀다고 하였다. 또 젊고 예쁜 여자들을 보면 은근히 부럽기도 하고 자신도 젊음을 위해 더 열심히 노력하고 싶다고 하였다. 그러므로 참여자들이 지금 할 수 있는 최선은 신체활동에 더 열심히 참여하는 것이라고 하였다. 또한 참여자들은 폐경이 되면서 쉽게 살이 쪄서 고민이며, 살이 찌면 관절도 아프고 더 늙어 보이기 때문에 조금이라도 체중이 늘어나면 바로 몸매관리를 위해 더 열심히 신체활동에 참여한다고 하였다. 더 늙기 전에 예쁜 옷도 입고 싶고, 살을 빼서 사람들이 날씬하다고 말해주면 기분이 너무 좋아지기 때문에 날씬한 몸매를 유지하기 위해 계속 더 노력하게 된다고 하였다.

“남들보다 5년, 10년 더 젊어 보이는 것이 얼마나 좋은 건지 몰라. 나이 먹으면 목욕탕에서도 자기보다 젊고 예뻐 보이는 여자들 보면 관심이 가고... 그러니까 조금이라도 주름이 보이면 바로 관리하지.” (참여자 4)

“외모가 자꾸 변하니까... 콜라겐… 뭐 그런 것 다 먹거든. 그런데도 탄력이 없는 거지. 너무 옛날하고 다르게 늙어가니까 그게 너무 싫은 거지. 그다음은 점점 더 늙는 것만 남아 있으니까 불안하고 젊음이 계속 신경 쓰이는 거지.” (참여자 8)

“내가 운동을 안 했으면 아마 엄청 아프고 살이 쪘을 걸요. 살찌면 관절이 아파서 걷지 못해요. 살이 찌니까 또 부랴부랴 운동해서 빼는 거지. 늙어가는 것도 서러운데 이런 데가 살이 찌면 더 늙어 보이고, 그러면 옷발 안 받아서 나갈 때마다 짜증나.” (참여자 4)

“살찌는 게 너무 싫어. 원래 살이 잘 찌는 체질이라 살이 자꾸 오르는데, 옷을 좀 마음에 드는 걸 입으려면 날씬해야 하니까 관리하기 위해서 또 운동을 해야지.” (참여자 8)

#### 주제 6: 몸과 마음 갖추기

참여자들에게 신체활동이란 곧 건강을 의미한다고 하였다. 참여자들은 더 곱고 우아하게 나이 들고 싶다고 하였다. 그리고 제발 아프지 않고 건강하게 살다가 죽을 때는 좋은 모습으로 죽을 수 있기를 희망하고 있었으며, 건강하게 나이 들기 위하여 더 열심히 신체활동에 참여한다고 하였다. 참여자들은 노년을 대비하여 보험을 든다는 생각으로 열심히 신체활동에 참여하고 있으며, 나이가 들어서도 할 수 있는 신체활동을 미리 배우고 있다고 하였다. 그리고 자기에게 가장 잘 맞는 신체활동을 인생의 선물이요 평생의 친구 같은 존재로 여긴다고 하였다.

“죽을 때 좋게 죽을 수 있게. 얼굴에 그 사람의 인생이 보인다고 하잖아요. 곱게 늙어야 한다고 생각해요. 곱고 고상하고 우아하게. 조금씩 많이 좋아지고 있어. 예전보다.” (참여자 3)

“운동은 곧 건강이지. 미를 떠나서 이 나이에 안 아프면 어디 가도 신체적인 조건이 되면 일할 수 있고, 경제적인 걸 얻을 수 있으니까 아프지만 말고 나이 먹었으면 좋겠어.” (참여자 5)

“나는 운동도 노년 대비로 하는 거야. 한 번 배워 놓으면 나만 채 들고 나가면 혼자 할 수도 있는 거고, 친구들하고 오래 걸을 수 있는 운동이니까 나이 먹어도 할 수 있어.” (참여자 4, 골다공증)

“운동은 인생의 보험이죠. 인생의 선물이고 친구 같은 존재예요. 요가는 내 인생의 마지막까지 가져갈 최후의 운동이고요. 아마 평생 안 외로울 것 같아요. 나는 하고 싶은 일 하면서 마지막까지 운동하고 그렇게 살 거예요.” (참여자 9)

이상과 같은 분석 결과를 통합한 폐경증상 관리를 위한 중년 여성의 신체활동 경험에 대한 최종 진술은 다음과 같다. 중년의 여성은 폐경증상으로 인해 소진된 몸과 마음을 회복하기 위해서 절박한 심정으로 신체활동을 선택했다. 신체활동에 참여하면서 몸과 마음이 점차 회복되기 시작했고, 그동안 자신을 옭아매고 있었던 남편에 대한 집착과 계속해서 쌓이는 스트레스의 굴레에서도 벗어나는 경험을 했다. 시행착오를 거쳐 자기에게 가장 잘 맞는 신체활동을 찾아내면서 그 재미에 푹 빠져들게 되었고, 점차 생활의 중심이 바뀌고 새로운 습관이 삶 속에 자리를 잡았다. 또한 오로지 자신에게 집중하는 시간을 가지면서 내적 성찰을 통해 있는 그대로의 자신을 사랑하게 되었고, 다른 사람을 이해하는 마음의 폭이 넓어지고 위축되었던 감정이 살아나는 등 자기를 찾고 오늘의 삶을 주도적으로 살아가게 되었다. 동시에 성큼성큼 다가오는 노화를 온몸으로 느끼면서 안간힘을 다해 신체활동에 더욱 매달렸다. 또한 건강하게 잘 나이 들고 싶은 바람과 함께 노년에도 지속할 신체활동을 미리 준비하면서, 몸과 마음을 갖추어 나가고 있었다. 이로써, 중년 여성은 폐경관리를 위해 신체활동에 참여하면서 과거의 고통에서 벗어나 오늘의 삶을 주도하고, 새로운 변화를 기대하며 계속해서 앞으로 나아가는 경험을 하는 것으로 확인되었다.

## Discussion

본 연구에서 폐경증상 관리를 위한 중년 여성의 신체활동 경험은 ‘과거의 고통에서 벗어나기’, ‘오늘의 삶에서 주도권 잡기’, ‘새로운 변화를 기대하며 나아가기’라는 본질적 의미가 있는 것으로 확인되었다. 분석된 주제묶음을 중심으로 본 연구의 결과를 다음과 같이 논의하고자 한다.

첫째, 폐경증상 관리를 위한 중년 여성의 신체활동 경험은 과거의 고통에서 벗어나기이다. 본 연구의 참여자들에게 과거의 고통이란 폐경증상으로 인한 고통, 대인관계에서 오는 갈등, 생활 속의 스트레스 등을 의미한다. 이것은 신체활동을 통해 이러한 고통에서 해방됨으로써 참여자들에게는 이미 지나간 과거로 인식되었음을 의미한다. 그리고 선행연구에서 보고한 폐경증상에 대한 신체활동의 완화 효과를[17-19] 본 연구의 참여자들도 경험하는 것으로 확인되었다. 참여자들은 자기에게 집중하게 되면서 다른 사람들에 대한 집착에서 벗어나는 경험을 하게 되었고, 그동안 쌓였던 스트레스를 신체활동을 통해 해소하는 것으로 나타났다. 이것은 신체활동을 통하여 돌봄의 대상을 ‘타자’에서 ‘나’로 전환하여 타인보다 자신에게 더 관심을 기울이게 되고[25], 긴장과 스트레스가 완화되는 경험에 대한 보고[20]와 유사하다고 할 수 있다. 또한 신체활동은 골다공증이 있는 폐경기 여성의 삶의 질에 영향을 미치는데[26], 본 연구의 참여자 중 폐경이 되면서 골다공증을 진단받은 참여자는 신체활동을 통해 골다공증으로 인한 만성통증이 개선되어 삶의 질이 올라간 것으로 확인되었고, 그 외 당뇨를 진단받은 참여자도 정상 혈당 수치를 유지하고 있는 것으로 확인되었다. 본 연구의 참여자들은 소득과 관계없이 가장 먼저 신체활동 프로그램에 등록한다고 하여 선행연구와 차이를 보였는데[5], 본 연구의 참여자들에게는 폐경증상의 고통에서 벗어나 남은 삶을 잘 살아내는 것이 가장 급선무였음을 시사하는 부분이다.

본 연구에서 참여자들은 폐경에 대해서는 자연스러운 노화의 과정으로 받아들이고 있었으나, 수면장애로 인한 고통을 많이 호소하였다. 또한 선행연구에서 이미 보고한 바와 같이[17] 참여자들은 폐경이 되면서 집에 있을 때에 우울감과 무력감이 더 심해지는 것을 경험했지만, 신체활동에 참여하면서 우울 수준이 많이 감소되었고 몸과 마음의 활력을 찾게 된 것으로 나타났다. 특별히 주목할 것은 신체활동에 참여하여도 폐경증상이 완전히 없어지지는 않았지만 폐경증상이 그전보다는 감소되었고, 남아 있는 증상도 신체활동에 참여함으로써 충분히 조절할 수 있었다는 점인데, 이는 참여자들이 폐경증상을 잊을 만큼 신체활동의 재미에 푹 빠지고, 자신의 인생을 스스로 통제할 수 있겠다는 자신감이 폐경증상의 조절에 영향을 미친 것으로 생각된다.

둘째, 폐경증상 관리를 위한 중년 여성의 신체활동 경험은 오늘의 삶에서 주도권을 잡는 것이다. 이는 본 연구의 참여자들이 스스로 자기 삶의 주도권을 잡고 변화하고자 하는 노력을 하며 현재에 집중하는 경험으로, 신체활동을 통한 몰입 경험이 본 연구에서도 확인되었다[21]. 참여자들에게 일상의 모든 영역에서 가장 우선적 선택은 신체활동에 참여하는 것이었는데, 그들에게 신체활동 시간은 깊은 자기성찰을 통해 그들의 삶을 주도적으로 이끌어갈 수 있는 힘을 얻는 기회가 되었다. 이는 선행연구에서도 신체활동을 통하여 중년 여성들이 정체성을 되찾을 뿐만 아니라 자신의 삶에서 주인공이 되는 새로운 경험을 하고[22], 능동적으로 자신의 삶을 새롭게 기획하고 주체적으로 재구성해 나가는 경험을 하는 것으로 보고하고 있어[25], 신체활동을 통하여 폐경기 여성들이 자기인식과 삶의 주체성을 회복하는 경험을 하는 것을 알 수 있다.

또한 폐경기 여성은 마음의 상태가 직접적인 몸의 증상으로 표출되기 쉽고 몸과 마음이 통합되는 느낌을 자주 경험한다는 보고와 같이[27] 본 연구의 참여자들도 폐경증상으로 인하여 몸과 마음이 함께 소진되고 위축되는 경험을 하였는데, 그들은 신체활동에 꾸준히 참여하면서 몸의 변화뿐만 아니라 마음이 안정되는 경험을 하게 되었고, 단지 센터에 들러 샤워만 하는 것으로도 마음의 위안을 얻기도 하였다. 30대–60대 성인여성을 대상으로 한 Park [25]의 보고에서도 신체활동 참여를 통하여 몸과 마음이 같이 바뀌면서 몸과 마음의 긴밀한 연결성에 대해 깨닫게 되는 경험을 하는 것으로 나타나, 규칙적인 신체활동이 위축된 몸과 마음을 유기적으로 변화시킨다는 것을 알 수 있다. 그러나 Park [25]은 여성의 생애경험에 주목하여 그룹운동 참여 경험을 밝히고자 하였으며, 이는 폐경증상 관리를 위한 경험에 초점을 둔 본 연구와 차이를 보인다. 또한 본 연구의 참여자들은 폐경기 여성들이 대인관계에서 경험할 수 있는 부정적인 변화를[10] 신체활동에 규칙적으로 참여하면서 극복한 것으로 확인되었다. 그들은 같은 여자로서 시어머니에 대해 측은지심이 생기게 되고 친정 어머니의 삶이 애잔하게 느껴지는 등 다른 사람에 대한 이해의 폭이 넓어지는 것을 경험하였는데, 이는 중년 여성들이 신체활동 참여로 인하여 마음이 안정되고 타인과의 관계가 원만해지고[20], 신뢰와 친밀감과 사회적 소통을 경험하며[21], 타인과의 교류와 관계 회복을 경험한다는 보고[22]와 맥을 같이 한다.

셋째, 폐경증상 관리를 위한 중년 여성의 신체활동 경험은 새로운 변화를 기대하며 나아가는 것이다. 이것은 삶이 더 나은 방향으로 변화하기를 기대하며 몸과 마음을 계속해서 갖추어 나가고자 하는 것으로, 폐경과 함께 찾아온 노화를 극복하고 젊음을 유지하기 위해 노력하는 것이 포함되는 의미이다. Yu와 Lee [20]는 요가 수행 경험을 몸과 마음이 변화하는 경험으로 보고하였으며, Yoon 등[22]은 라인댄스 참여 경험을 자기를 찾아가는 전환점에 대한 기대감으로 제시하였는데, 이는 본 연구와 일맥상통하는 부분이라고 할 수 있다. 또한 본 연구의 참여자들은 폐경을 늙어가는 것으로 인식하고 있었고, 늙어간다는 것에 대한 심리적 불안감을 느끼기도 하였다. 대체로 폐경기 여성들은 노화를 경험하며 건강관리를 더욱 결심하는 경향이 있는데, 선행연구에서는 이를 영성을 통하여 극복한 것으로 나타나[28] 본 연구와 차이를 보인다. 본 연구의 참여자들은 신체활동을 통해 젊음을 유지하려고 노력하였는데, 이것은 몸은 늙어가고 있지만 젊음에 대한 열정과 기대는 시간이 지나도 시들지 않는 중년 여성의 심리를 잘 나타내 주고 있으며, 이러한 심리는 때로 신체활동에 과도하게 집착하는 것으로 나타나기도 하였지만, 참여자들은 오히려 이런 집착이 신체활동을 지속하게 하는 것으로 인식하고 있었다.

본 연구에서 폐경기 여성에게 나타나는 노화에 대한 불안은[11,12] 노년에도 실천할 수 있는 신체활동을 미리 구상하면서 노후를 준비하는 것으로 표출되었는데, 대부분의 참여자들은 노년을 위한 신체활동으로 요가를 지목하였다. 요가는 중년 여성의 만성통증에 효과가 있는 등[18] 지금까지 40대–50대 여성을 대상으로 하여 활발한 연구가 진행되어 왔으며 60대 이상의 여성에서도 점차 관심이 높아지고 있는 실정이다[29]. 선행연구에서는 요가 수행 경험을 ‘삶, 그 자체’로 회복과 성장을 통해 온전한 삶으로 살아가는 경험으로 보았는데[20], 본 연구의 참여자들도 신체활동을 곧 삶으로 인식하고 있었고 계속해서 긍정적인 삶의 변화를 이끌어내고자 노력하는 것으로 확인되었다. 또한 운동 관련 지각된 유익성이 높을수록 운동 수행 정도는 높고[30], 참여 만족은 운동 지속에 영향을 주게 되는데[31], 폐경기에 해당하는 본 연구의 참여자들의 경우에는 폐경증상의 많은 부분이 신체활동을 통해 개선될 뿐만 아니라 재미와 함께 건강이 점점 더 좋아질 것이라는 긍정적 기대감이 신체활동을 지속하게 하는 것으로 보였다.

이상과 같이 폐경기 여성은 신체활동을 통하여 폐경증상, 관계, 스트레스 등을 극복하고 삶에서 긍정적인 변화를 경험하며 앞으로의 삶에 대한 기대감을 갖게 된다. 본 연구는 폐경증상 관리를 위한 신체활동의 실제 경험을 확인함으로써 참여자들의 관점에서 그들의 경험을 이해할 수 있다는 데 의의가 있다. 본 연구가 폐경기 여성의 고통과 경험을 좀 더 깊이 이해하고 폐경기 관리를 위한 신체활동 참여를 높일 수 있는 중재 전략 개발의 실질적인 기초 자료로 제공될 수 있기를 기대한다. 또한 앞으로 폐경증상을 중점적으로 관리하도록 돕는 신체활동 프로그램이 많이 보급되고, 폐경기 여성만을 전담으로 하는 지역 시스템이 구축되어 활성화되어야 할 것이다. 아울러 본 연구에서는 시간의 경과에 따른 경험을 구체적으로 이해하기에는 제한이 있으므로, 관심 현상에 대한 이해의 폭을 넓히기 위하여 추후에는 종적 연구를 통해 폐경기 신체활동 경험의 구체적인 과정에 대해 좀 더 살펴볼 필요가 있다.

## Figures and Tables

**Table 1.** Characteristics of participants (N=9)

ID	Age (year)	Age at menopause (year)	Occupation	Menopausal symptoms	Physical activity		
					Type	Duration	Frequency of >50 min/week
1	58	48	Housewife	Hot flashes, sleep disorder, digestive disorder, stress	Zumba	1 year	3
					Swimming	10 years	5
2	56	50	Social worker	Hot flashes, depression, stuffy in the chest, sleep disorder, fatigue	Valley	2 years	3
					Yoga	2 years	3
3	52	49	Housewife	Hot flashes of the sole, frozen shoulder, palpitations during sleep	Yoga	2 years	5
4	59	50	Housewife	Dry skin, lumbar pain, emotional ups and downs, frozen shoulder	Zumba	1 year	3
					Yoga	1 year	3
					Golf	1 year	3
5	57	53	Sales position	Hot flashes, sleep disorder	Yoga	4 months	3
6	55	54	Self-employment	Vaginal drying, cystitis, emotional ups and downs	Aerobics	2 years	5
7	59	55	Hairdresser	Vaginal pain, diminished sexual desire, irritability	Aerobics	8 years	5
8	55	54	Company staff	Depression, irritability, cold sweats	Zumba	3 years	3
					Yoga	3 years	3
9	52	50	Housewife	Hot flashes, depression, sleep disorder, headache	Yoga	4 years	3

**Table 2.** Codes, themes, and theme clusters of the meaning of physical activity for menopausal symptom management

Code	Theme	Theme cluster
Living with an exhausted body	Reviving the exhausted body and mind	Overcoming my past pain
A gradual improvement in menopausal symptoms		
Free from the obsession with relationships	Being free from the yoke of pain	
Relieving accumulated stress		
Finding the right one	Being settled in life	Taking the initiative for today’s life
In love with fun		
Being the center of life		
Time for only me	Finding oneself and becoming altruistic	
Recovered emotions		
Broadened mind		
A desire to be young	Striving while anticipating change	Moving towards new change
Losing weight in a hurry		
Aging healthy	Equipping the body and mind	
Preparing for old age		
